# EasyCGTree: a pipeline for prokaryotic phylogenomic analysis based on core gene sets

**DOI:** 10.1186/s12859-023-05527-2

**Published:** 2023-10-14

**Authors:** Dao-Feng Zhang, Wei He, Zongze Shao, Iftikhar Ahmed, Yuqin Zhang, Wen-Jun Li, Zhe Zhao

**Affiliations:** 1 Jiangsu Province Engineering Research Center for Marine Bio-resources Sustainable Utilization and College of Oceanography, Hohai University, Nanjing, 210098 China; 2 Key Laboratory of Marine Biogenetic Resources, Third Institute of Oceanography, Ministry of Natural Resources, Xiamen, 361005 China; 3 National Agricultural Research Centre (NARC), Land Resources Research Institute (LRRI), National Culture Collection of Pakistan (NCCP), Islamabad, 45500 Pakistan; 4 Institute of Medicinal Biotechnology, Chinese Academy of Medical Science and Peking Union Medical College, Beijing, 100050 China; 5 State Key Laboratory of Biocontrol, Southern Marine Science and Engineering Guangdong Laboratory (Zhuhai) and Guangdong Provincial Key Laboratory of Plant Resources, School of Life Sciences, Sun Yat-Sen University, Guangzhou, 510275 China

**Keywords:** Phylogeny inference, Supermatrix, Supertree, Prokaryote taxonomy, Core gene

## Abstract

**Background:**

Genome-scale phylogenetic analysis based on core gene sets is routinely used in microbiological research. However, the techniques are still not approachable for individuals with little bioinformatics experience. Here, we present EasyCGTree, a user-friendly and cross-platform pipeline to reconstruct genome-scale maximum-likehood (ML) phylogenetic tree using supermatrix (SM) and supertree (ST) approaches.

**Results:**

EasyCGTree was implemented in Perl programming languages and was built using a collection of published reputable programs. All the programs were precompiled as standalone executable files and contained in the EasyCGTree package. It can run after installing Perl language environment. Several profile hidden Markov models (HMMs) of core gene sets were prepared in advance to construct a profile HMM database (PHD) that was enclosed in the package and available for homolog searching. Customized gene sets can also be used to build profile HMM and added to the PHD via EasyCGTree. Taking 43 genomes of the genus *Paracoccus* as the testing data set, consensus (a variant of the typical SM), SM, and ST trees were inferred via EasyCGTree successfully, and the SM trees were compared with those inferred via the pipelines UBCG and bcgTree, using the metrics of cophenetic correlation coefficients (CCC) and Robinson–Foulds distance (topological distance). The results suggested that EasyCGTree can infer SM trees with nearly identical topology (distance < 0.1) and accuracy (CCC > 0.99) to those of trees inferred with the two pipelines.

**Conclusions:**

EasyCGTree is an all-in-one automatic pipeline from input data to phylogenomic tree with guaranteed accuracy, and is much easier to install and use than the reference pipelines. In addition, ST is implemented in EasyCGTree conveniently and can be used to explore prokaryotic evolutionary signals from a different perspective. The EasyCGTree version 4 is freely available for Linux and Windows users at Github (https://github.com/zdf1987/EasyCGTree4).

**Supplementary Information:**

The online version contains supplementary material available at 10.1186/s12859-023-05527-2.

## Background

Phylogenetic analysis uses genome-based methods more and more routinely rather than a small number of genes, for interpreting the evolutionary and genetic information of prokaryotes [1, 2]. There are two main approaches most frequently used in inferring phylogenies from large gene collections [2, 3]. The supermatrix (SM) uses gene concatenation to reduce stochastic errors, and allow the combination of weak phylogenetic signals in different genes. The supertree (ST) derives the optimal tree obtained through the analysis of individual genes of interest that is unnecessary to be present in every genome. This approach prevents the combination of genes with incompatible phylogenetic histories [4]. The ST can be easily parallelized in practice and does not require as much memory as the SM. They are originally developed by replacing phylogenies based on a limited number of house-keeping genes, which have been widely used historically for characterizing the taxonomy, evolution, and genotypic characterization of prokaryotes and constitute the general framework [2]. In the genomic era, the number of house-keeping genes covered in phylogenies of prokaryotes has increased, such as the *rps* gene set (53 genes encoding the bacterial ribosome protein subunits) [5], bac120/ar122 gene set [6], and up-to-date bacterial core gene (UBCG) set [7]. Furthermore, core gene sets based on pan-genome have been defined for phylogenetic analysis within different taxonomic ranks, particularly from species to family [8].

However, the considerable bioinformatics skills needed in analyzing a large volume of genomic data and the complex formats of data from different applications impedes related analysis by beginners. Detecting the core genes of a customized genome data set is time consuming and requires a powerful machine. Several tools have been developed for phylogenomic analysis. The autoMLST pipeline scansconserved single-copy housekeeping genes, and builds a phylogeny by using SM (concatenated gene matrix) or ST (coalescent tree with ASTRAL-III) [9]. The UBCG pipeline uses a gene set, named UBCG, to build an SM phylogeny [7], and GToTree estimates genome completeness and redundancy, and infers an SM phylogenomic tree according to a gene set of custom hidden Markov models (HMMs) or one of its 13 enclosed HMMs [10]. The bcgTree pipeline extracts 107 core genes (included in the essential gene set) by using HMMs and performs a phylogenetic analysis with SM [11]. All these tools can be employed by Linux users only, and specific libraries and third-party software are required for their installation. These requirements are prohibitive for Windows users and biologists not focusing on bioinformatics.

In this study, we introduced EasyCGTree, which is a user-friendly and cross-platform Perl-language (https://www.perl.org/) tool, for constructing genome-scale maximum-likehood (ML) phylogenetic tree with SM and ST. It uses microbial genomic data (amino acid sequence) as input data, and the profile HMMs of core gene sets for homolog searching. It is an all-in-one automatic pipeline from input data to phylogenomic tree, is highly portable, and can be operated on a personal computer or powerful server running either Linux or Windows.

## Implementation

EasyCGTree was implemented in Perl programming languages (https://www.perl.org/) and was built using a collection of published reputable tools, including Clustal Omega version 1.2.4 [12]; consense from PHYLIP version 3.698 [13]; FastTree version 2.1 [14]; hmmbuild and hmmsearch from HMMER version 3.0 (http://hmmer.org/); IQ-TREE version 2.1.1 [15]; trimAl version 1.2 [16]; and wASTRAL version 1.15.2.3 [17]. Most tools required by EasyCGTree were precompiled as standalone executable files and contained as a single package. It can run after the installation of Perl language environment. It supports the Linux 64-bit architecture and Windows version 7 and above. Experienced users can replace the included tools with precompiled up-to-date versions easily to update EasyCGTree, and we will update these tools and the main scripts aperiodically to ensure longevity. It will be tried to develop a version on MAC OS that is as portable as those on Windows and Linux.

**Fig. 1:**
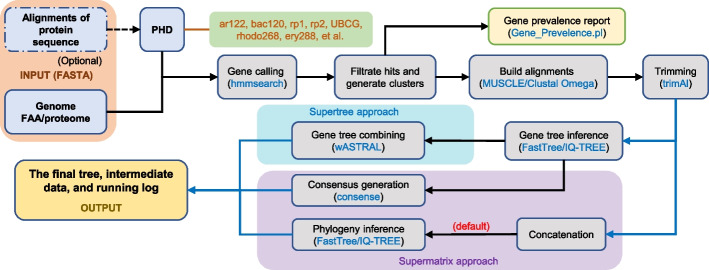
EasyCGTree flowchart. *Note:* The algorithm uses FASTA/multi-FASTA formatted amino acid sequences from prokaryotic genomes (i.e., proteome) as input data. EasyCGTree includes the supermatrix (SM) and supertree (ST) approaches to infer phylogeny. Several profile HMMs of core gene sets have been prepared in advance to construct a profile HMM database (PHD) that is enclosed in the package and used for homolog searching. Customized gene sets can also be used to build profile HMMs and added to the PHD

Several profile HMMs of core gene sets were prepared in advance for the construction of a profile HMM database (PHD) enclosed in the package and used for homolog searching with HMMER (http://hmmer.org/). Customized gene sets (prepared as gene clusters) can be used to build profile HMMs by EasyCGTree and added to the PHD. Currently, the PHD comprises the following gene sets: bac120, 120 ubiquitous genes (corresponding to 120 protein domains) in the domain *Bacteria* [18]; ar122, 122 ubiquitous genes (122 protein domains) in the domain *Archaea* [18]; rp1, 16 ubiquitous ribosomal protein genes (18 protein domains) in *Prokaryote* [19]; rp2, 23 ubiquitous ribosomal protein genes (27 protein domains) in *Prokaryote* [20]; UBCG, 92 up-to-date bacterial core genes in *Bacteria* [7]; ery288, 288 core genes of the family *Erythrobacteraceae* [21]; and essential, 107 essential single-copy core genes in *Bacteria* [11]. EasyCGTree can facilitate core-gene based phylogeny inference and is portable. Furthermore, the intermediate data of an EasyCGTree run can be directly used as input data of many other applications. An overview of the workflow is shown in Fig. 1, and more details can be found in the EasyCGTree package manual.

### Input

EasyCGTree uses FASTA/multi-FASTA-formatted amino acid sequences from prokaryotic genomes (i.e., proteome) as input (option-proteome). The file names of the proteomes will be formatted automatically in order that the labels can be processed correctly. The command line will be checked for validity before a run, including the file number in the input directory (≥ 5); sequence type (protein sequence) and formation (FASTA-formatted); and consistency among options. Error reports will be sent back to users if some options are set invalidly. Otherwise, a run will start, and a run log will be printed on the screen and will be saved in a log file named according to the name of the input directory and local starting time.

### Gene calling

An HMM file of a gene set can be specified by using the option “-hmm” (default bac120) and can be used in searching homologs against each proteome with hmmsearch from the HMMER package (http://hmmer.org). An E value 1e-10 can be used as the default threshold for HMM searching, and it can be modified by using the option “-evalue”.

### Filtrate hits and generate clusters

The top hit of each gene was screened according to the E value threshold. Genomes with fewer gene detected and genes with low prevalence will be excluded by applying options “-genome_cutoff” and “-gene_cutoff”, respectively. Subsequently, the homologs of the selected genes were retrieved from the selected proteomes and prepared as gene clusters. A gene prevalence report can be generated by using the Perl script “Gene_Prevelence.pl” from the EasyCGTree package.

### Build alignments

MUSCLE [22] was used for multiple sequence alignment in Windows to ensure accuracy, and Clustal Omega [12] was used in Linux. The reasons were that: Clustal Omega is faster than MUSCLE for extremely large alignments, and Linux is preferred in powerful servers. Experienced uses can employ other pre-compiled tools for alignment via the modification of several lines of code in the EasyCGTree script.

### Trimming

The tool trimAl [16] was used for alignment trimming and conserved segment selection, and three different automatic methods (i.e. gappyout, strict and strictplus) were implemented using trimAl for the selection of different thresholds on the basis of MSA features. A standard for trimming alignment used by trimAl can be set by using the option “-trim” (default strict). The strict method combines a gappyout trimming with a subsequent trimming based on an automatically selected similarity threshold. More information can be found in the trimAl manual (http://trimal.cgenomics.org/trimal).

### Phylogeny inference

SM and ST [1, 2] can be set using the option “-tree” (default supermatrix; -tree sm). The two tree-inference programs, namely FastTree [14] and IQ-TREE [15], can be specified by using the option “-tree_app” (default FastTree). FastTree was used as the default mainly for the consideration on initial completeness of phylogenomic analysis because of its faster speed and less requirement of memory. IQ-TREE was highly recommended for its accuracy on powerful machines or with a small input dataset [23]. If SM is used, a concatenation of each trimmed alignment will be generated and subjected to infer phylogeny with a selected program. If a proteome is missing in a gene cluster, it will be treated as gaps at related segment of the concatenation. If ST is used (-tree st), gene trees will be inferred from each trimmed alignment of the gene clusters (do not require all taxa to be present), and then the program wASTRAL [17] will be employed to combine them into a single ST with the hybrid method that consider phylogenetic uncertainty by integrating signals from branch length and branch support in gene trees.

In addition, the classic consensus technique (e.g., majority rule consensus tree; -tree cs) [24] was included in EasyCGTree to explore consistency among core gene trees and can be regarded as a variant of the typical SM with concatenation [1]. For this approach, the option “-gene_cutoff” will be set as “1” to ensure that each taxon would be present in all the gene trees inferred from the trimmed alignment of each gene cluster. Then the program consense [13] will be used to generate a consensus tree of the type “Majority rule (extended)”. This type of consensus tree uses the following principles: any set of taxa will be included if it appears in more than 50% of the gene trees; the other sets of taxa are considered in the order of the frequency of their appearance, and added to the consensus tree compatible with it; all the taxa will be added to the consensus tree until the tree is fully resolved.

Options within FastTree and IQ-TREE can be changed by editing the file “tree_app-options.txt” attached with the EasyCGTree package. Notably, the specified tree-inference program determines the maximum memory required by EasyCGTree. Users are encouraged to refer to the documentations of FastTree and IQ-TREE to evaluate whether their machines can run EasyCGTree successfully. If it cannot be run by a machine, small input data set, gene set with few genes, and a powerful machine should be considered.

### Outputs

All the data generated during a run were recorded, including HMM searching result, sequence of gene clusters, alignment, gene tree, and running log. The final phylogenetic tree was written in Newick format. Users can display it via FigTree (http://figtree-international.com/), MEGA [25], iTOL [26] or other tree viewers.

### Profile HMM database (PHD)

For the ar122, bac120, essential, rp1, rp2, and UBCG gene sets, the accession numbers of the genes included in each gene set were retrieved from previous reports [7, 11, 18–20]. All the HMMs were downloaded (on July 7, 2022) from the Pfam-A (www.pfam.org/) and NCBI (https://ftp.ncbi.nih.gov/hmm/) HMM databases. Subsequently, the HMM of each gene was retrieved from the local HMM databases and merged into a single file according to the accession list of each gene set. For the ery288 gene set, the alignments of each gene cluster were retrieved from our previous study [27] and built with the Perl script “BuildHMM.pl” from the EasyCGTree package. Customized gene sets prepared as gene clusters can also be used in building profile HMMs and added to the local PHD via this script.

## Results and discussion

### Examples in publications

EasyCGTree has been used and cited in some publications, most of which are studies on novel bacterial taxa descriptions. Phylogenies inferred with EasyCGTree were compared with those in reports associated with closely related taxon, and similar topologies were observed between them. The current taxonomic framework of the family *Erythrobacteraceae* was established mainly by using phylogeny based on 288 core genes [21] and was named ery288 in this study. With ery288 and the substitution model LG + F + R9 for IQ-TREE as the previous report (with manually controlled workflow) [21], an SM phylogeny with an identical topology was recovered by using EasyCGTree [28]. Most genera of this family can be recovered as monophyletic groups in the phylogeny of ery288 inferred with FastTree employed by EasyCGTree, except the genera *Alteriqipengyuania*, *Croceibacterium*, and *Qipengyuania* [27]. Although low accuracy was observed, EasyCGTree employing FastTree (< 40 min) had an advantage over employing IQ-TREE (> 12 h) in terms of elapsed time when a personal computer (Intel Core i7-9700 CPU and 16 Gb RAM) running Windows 10 was used.

The consensus approach in EasyCGTree was successfully applied in clarification of the relationships between the genera *Marmoricola* and *Nocardioides* [29], and in the classification of strain HHU G3-2 as a new species of the genus *Aestuariicella* [30]. However, there is rare case of applying ST approach in prokaryotic study, and we cannot perform further validation of ST implemented in EasyCGTree. In a recent study, we applied the EasyCGTree package to build the HMMs of the genes involved in the ammonification metabolism pathway, and the prevalence of related gene families in genus *Alteromonas* has been extensively characterized [31].

### Performance compared with other tools

A comparison of the features of EasyCGTree and other tools with similar functions mentioned above is summarized in Table 1. EasyCGTree includes third-party software also used by others: trimAl [16] is also employed by autoMLST [9] and GToTree [10]; MUSCLE [22] also by GToTree [10] and bcgTree [11]; FastTree [14] by UBCG and GToTree [10]; and IQ-TREE [15] by autoMLST [9]. The highlighted advantages of EasyCGTree are that it supports Window and does not require preinstalled software. Thus, it is easier to use than other pipelines.

**Table 1:** A comparison of the features among EasyCGTree and other pipelines with similar functions

Pipeline	EasyCGTree	autoMLST	bcgTree	GToTree	UBCG
Operation system	**Linux, Windows**	Linux	Linux	Linux	Linux, Mac OS
Lanuage	Perl	Java, Python	Java, Perl	Python	Java
Additional module	**None**	None	Five Perl modules	None	None
Package size	17 MB (Linux); 20 MB (Windows)	~ 25 GB	17 MB	140 KB (without HMMs)	8 MB
Prerequisites	**Perl**	conda, Python, git	git, Java, Perl	conda, Python	Java
Pre-installed software	None*	hmmsearch, trimAl, ASTRAL, IQ-TREE, MAFFT, MASH, Prodigal, RaxML	hmmsearch, Gblocks, MUSCLE, Prodigal, RaxML	hmmsearch, trimAl, FastTree, MUSCLE, Prodigal, TaxonKit	hmmsearch, FastTree, MAFFT, Prodigal
Default HMM	bac120	Depends on bacterial family	essential	Not specified	UBCG
HMM extensibility	**Yes**	No	Yes	Yes	No
Input	Prot	Nucl	Nucl, prot	Nucl	Nucl
Trimming	trimAl	trimAl	Gblocks	trimAl	None
Alignment	MUSCLE or Clustal Omega	MAFFT	MUSCLE	MUSCLE	MAFFT
Tree-making approach	**SM, ST**	SM, ST	SM	SM	SM
Phylogeny inference	FastTree, IQ-TREE	IQ-TREE, RaxML	RaxML	FastTree	FastTree, RaxML

All of them use hmmsearch (HMMER, http://hmmer.org) for homolog searching

HMM, hidden Markov model; SM, supermatrix; ST, supertree

The advantages of EasyCGTree are marked in bold

*All the third-party programs are enclosed in the EasyCGTree package

Subsequently, we conducted phylogenomic analysis of the genus *Paracoccus* by using EasyCGTree and two other easy pipelines (UBCG version 3.0 [7] and bcgTree version 1.2.0 [11]) to evaluate their performance on a Dell PowerEdge T430 sever (2 × Xeon E5-2680 v4 CPU, 128 GB RAM) running Ubuntu 18.04.4 LTS. GToTree [10] was not included because the links for downloading the HMMs were broken, and autoMLST [9] was not included because it was not portable enough (with databases ~ 25 GB). A total of 43 genomic datasets of the genus *Paracoccus* and an outgroup were downloaded from the RefSeq database on the NCBI server (https://www.ncbi.nlm.nih.gov/; Additional file 1: Table S1).

**Fig. 2:**
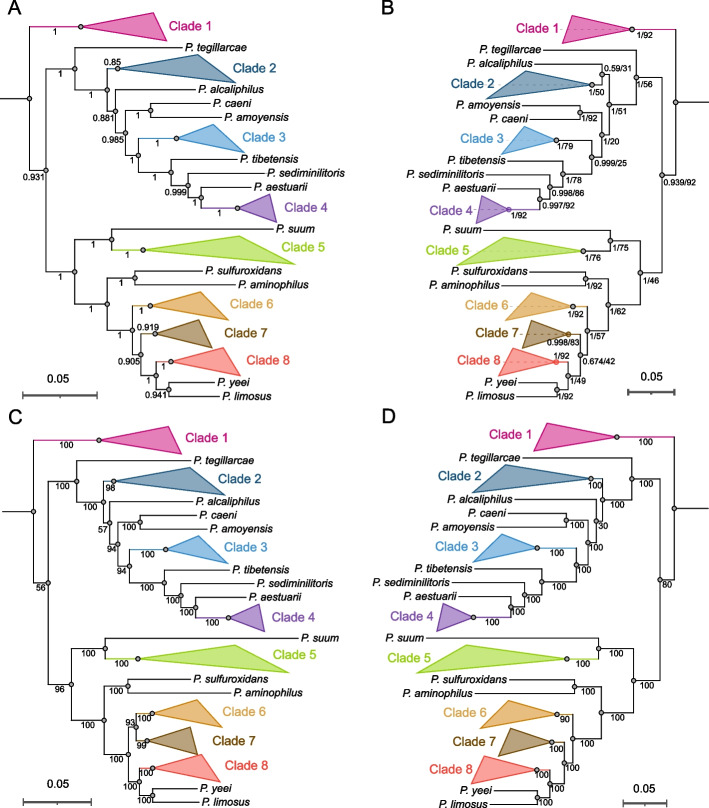
Phylogenomic trees of the genus *Paracoccus* constructed using the pipelines EasyCGTree, UBCG, and bcgTree. *Note:* **A** EasyCGTree using 90 genes of the UBCG gene set (gene families TIGR03625 and TIGR01044 were excluded because of low prevalence) as the HMM and other default options. **B** UBCG using default options. **C** EasyCGTree using the essential gene set as the HMM and IQ-TREE for phylogeny inference with best-fit model Q.yeast + F + I + I + R5. **D** bcgTree using default options. Clades with identical topology among the four trees are collapsed and marked in the same color. Support confidence (A, 0–1; B, 0–1/≤ 0–92; C and D, 0–100) is indicated in the middle of branches or near the nodes. Support values in A and the former of those in B are calculated by using the Shimodaira-Hasegawa test with FastTree, and later of those in B are the gene support indices (GSI, the gene trees supporting the clade) generated by the UBCG pipeline. Support values in C and D are standard bootstraps generated by IQ-TREE and RaxML, respectively. Support values, > 0.7 for A and B, 64 for B (> 70% out of the 92 gene trees), and 70 for C and D, are considered as significant. All the trees were rooted at the outgroup *Roseobacter litoralis* Och 149 (GCF_000154785.2), which was omitted. Bar, 0.05 substitutions per amino acid position

As shown in Fig. 2, the SM tree inferred using EasyCGTree with default options (except that the UBCG gene set as the HMM was used) had a similar topology with stronger support (support values > 0.85) than that (support values > 0.59) obtained with the UBCG pipeline with default options (Additional file 1: Table S2). In particular, *P. alcaliphilus* formed a later branching lineage (support value 0.88) of the genus than Clade 2 in one tree (Fig. 2A) but closely neighboured Clade 2 with low-level confidence (support value 0.59) in the other tree (Fig. 2B). We checked the concatenations produced via the EasyCGTree and UBCG pipelines, and found that they included 26 089 and 30 232 amino acid positions, respectively. The appearance of differences in topology and confidence may depend on whether a trimming method was applied (Additional file 1: Table S2). This suggested that EasyCGTree can infer more reliable phylogeny than the UBCG pipeline. With regard to elapsed time, EasyCGTree took 6.5 min for the run, whereas the UBCG pipeline took 18 min. It was notable that the first step of the UBCG run (converting genome sequence to bcg files one by one manually) accounted for nearly 16 min. No convenient method was provided with the UBCG pipeline to help users at this laborious step [7]. Thus, the UBCG pipeline is unsuitable for handling hundreds of input datasets.

We used IQ-TREE in EasyCGTree to build a conduct phylogenomic tree of the genus *Paracoccus* on the basis of the essential gene set, and compared the tree with that made via bcgTree [11] using default options (Fig. 2C, D; Additional file 1: Table S2). The trees had nearly identical topologies with nodes most of which were well-supported (bootstrap > 90), except that Clades 6 and 7 clustered together in Fig. 2C, and Clade 7 clustered with Clade 8 and another two species and then with Clade 6 in Fig. 2D. In particular, *P. alcaliphilus* was placed in a position identical to that in Fig. 1A, but it was not well-supported in both trees (bootstraps 57 and 30, respectively). EasyCGTree and bcgTree used different methods for alignment (Clustal Omega [12] vs. MUSCLE [22]), trimming (trimAl [16] vs. Gblocks [32]), and phylogeny inference (IQ-TREE [15] vs. RaxML [33]) (Additional file 1: Table S2). This result suggested that EasyCGTree can conduct reliable phylogenomic analysis as bcgTree. EasyCGTree took 4 h and 44 min (including best-fit model selection), whereas the bcgTree pipeline took 12 h and 14 min. When 50 threads are used instead, EasyCGTree and bcgTree only took 31 min and 1 h and 53 min, respectively. EasyCGTree had considerably higher efficiency than bcgTree because IQ-TREE [15] is much faster than RaxML [33].

To further compare phylogenetic topologies among the four trees, we calculated pairwise cophenetic correlation coefficients (CCCs) and topological distance (i.e., Robinson-Foulds distance, RF) from the Newick files with the dendextend [34] and ape (http://ape-package.ird.fr/) packages in R v4.2.2 (https://github.com/rstudio/rstudio), respectively (Table 2). The CCC facilitates the calculation of the correlation between two cophenetic distance matrices of the two trees, and the value can range from − 1 to 1. Values near 1 mean that the two trees are nearly identical. The CCC values of > 0.99 suggested that EasyCGTree can produce nearly identical trees with similar methods conducted by UBCG and bcgTree. Compared with the tree from the UBCG and essential gene sets, the CCC decreased inapparently (0.957–0.982; Table 2). The RF distance is originally defined as twice the number of internal branches defining different bipartitions of the tips [35]. The branch length score used in this study is similar to the previous distance but considers branch lengths [36]. The results suggested the limited topological distance among the four trees (< 0.1; Table 2), although we were unable to summarize some rules as we did for the CCC analysis. Overall, these results indicated that EasyCGTree can construct SM trees with topologies comparable to those of UBCG and bcgTree.

**Table 2:** Pairwise cophenetic correlation coefficients (CCC) and Robinson-Foulds distance (RF) among supermatrix (SM) trees in this study

Method	EasyCGTree (UBCG)	UBCG	EasyCGTree (essential)	bcgTree
EasyCGTree (UBCG)	–	0.0556	0.0225	0.0962
UBCG	0.9923	–	0.0534	0.0468
EasyCGTree (essential)	0.9794	0.9577	–	0.0902
bcgTree	0.9929	0.9815	0.9915	–

The CCC values are below diagonal, while the RF distance are above diagonal. The method details and corresponding trees could be found in Fig. 2: EasyCGTree (UBCG), Fig. 2A; UBCG, Fig. 2B; EasyCGTree (essential), Fig. 2C; and bcgTree, Fig. 2D

In addition, EasyCGTree was used in constructing consensus tree and ST of the genus *Paracossus* (Additional file 1: Figure S1). We failed to assess the accuracy of the two approaches, because no portable tool that can perform similar analysis is available. The CCC and topological distance analysis cannot be analysed because the two trees lack of normal branch length that can be considered. However, the consensus and ST trees (Additional file 1: Figure S1) had topologies similar to those of the four SM trees in Fig. 2, although Clades 6 and 7 were divided and *P. alcaliphilus* clustered with *P. amoyensis* and *P. caeni* in the ST tree, which was not observed in the other trees. This finding suggested that ST constructed by EasyCGTree also accounts for prokaryotic phylogenomic analysis as it may draw evolutionary signals from a different perspective, although they were not widely used in prokaryotes currently. Similar to SM, ST is regarded as a critical way to corroborate an evolutionary hypothesis and to infer species tree. We expected ST to be beneficial for recent or rapidly diverging lineages of prokaryotes, as documented for eukaryotes [1, 9, 24, 37].

On the basis of results from the six trees (Fig. 2; Additional file 1: Figure S1), the evolutionary positions of most clades (Clades 1–5) and lineages were regarded as well resolved in the genus *Paracoccus*, because their positions were consistent among at least five trees (83%) and most of the support values were significant (> 0.7, > 70, or > 64 gene trees). Nevertheless, the positions of *P. alcaliphilus* and species in Clades 6–8 needed further clarification with enhanced methods or more reasonable gene sets, because low support values and low level of agreements (≤ three trees) were determined among the trees in this study.

## Conclusions

We presented the portable, flexible, and cross-platform tool EasyCGTree for genome-based phylogenetic tree reconstruction with SM and ST. Compared with other tools, EasyCGTree was much easier to install and use, and the robustness and accuracy were guaranteed. This tool will benefit microbiologists, especially individuals who use a computer running Windows or do not have a focus of bioinformatics.

## Availability and requirements

Project name: EasyCGTree

Project home page: https://github.com/zdf1987/EasyCGTree4

Operating system(s): Linux, Windows

Programming language: Perl

Other requirements: Perl 5.0 or higher

License: GNU GPL

Any restrictions to use by non-academics: license needed

### Supplementary Information

**Additional file 1: Table S1** Genomic information of strains used for phylogenomic analysis in this study; **Table S2** Performance of EasyCGTree, UBCG, and bcgTree conducting phylogenomic analysis of the genus *Paracoccus*; **Figure S1** Consensus tree and supertree (ST) of the genus *Paracossus* from the gene set UBCG with EasyCGTree.

## Data Availability

EasyCGTree version 4 is freely available at GitHub (https://github.com/zdf1987/EasyCGTree4). All information regarding installation and application of the pipeline is provided.

## Abbreviations

HMM: Hidden Markov model
ML: Maximum-likehood
PHD: Profile HMM database
SM: Supermatrix
ST: Supertree
UBCG: Up-to-date bacterial core gene
