# Supplementary material for: EasyCGTree: a pipeline for prokaryotic phylogenomic analysis based on core gene sets
Source: BMC Bioinformatics. 2023 Oct 14;24:390. doi: 10.1186/s12859-023-05527-2 (PMC10576351; doi:10.1186/s12859-023-05527-2)
Supplement: Supplementary file 1 — Additional file 1: Table S1 Genomic information of strains used for phylogenomic analysis in this study; Table S2 Performance of EasyCGTree, UBCG, and bcgTree conducting phylogenomic analysis of the genus Paracoccus; Figure S1 Consensus tree and supertree (ST) of the genus Paracossus from the gene set UBCG with EasyCGTree. [file 12859_2023_5527_MOESM1_ESM.pdf]

Table S1 Genomic information of *Paracoccus* strains used for phylogenomic analysis in this study

No	Species	Type strain	Size (Mb)	GC content (%)	Asembly
1	*P. acridae*	CGMCC 1.15419	3.99	65.3	GCA_014642735.1
2	*P. aeridis*	JC501	3.32	69.4	GCA_004923205.2
3	*P. aerius*	KCTC 42845	4.17	65.0	GCA_016757275.1
4	*P. aestuarii*	DSM 19484	3.75	67.7	GCA_003594815.1
5	*P. aestuariivivens*	NBRC 111993	4.56	61.4	GCA_009711225.1
6	*P. alcaliphilus*	DSM 8512	4.61	64.3	GCA_900110285.1
7	*P. alkanivorans*	44653	4.66	62.1	GCA_003697785.1
8	*P. alkenifer*	DSM 11593	3.19	67.3	GCA_900108405.1
9	*P. aminophilus*	JCM 7686	4.87	63.4	GCA_000444995.1
10	*P. aminovorans*	DSM 8537	3.95	67.5	GCA_900113565.1
11	*P. amoyensis*	11-3	3.73	60.1	GCA_014490725.1
12	*P. caeni*	MJ17	4.19	62.2	GCA_016629665.1
13	*P. chinensis*	CGMCC 1.7655	3.63	68.1	GCA_900102885.1
14	*P. denitrificans*	DSM 413	5.19	66.8	GCA_900100045.1
15	*P. endophyticus*	SYSUP0003	3.20	69.8	GCA_003286075.1
16	*P. fontiphilus*	MVW-1	4.11	66.2	GCA_017356265.1
17	*P. halophilus*	CGMCC 1.6117	4.01	65.2	GCA_900111785.1
18	*P. homiensis*	DSM 17862	3.87	63.8	GCA_900111675.1
19	*P. isoporae*	DSM 22220	3.52	65.8	GCA_900101865.1
20	*P. laeviglucosivorans*	DSM 100094	4.30	63.1	GCA_900182695.1
21	*P. liaowanqingii*	2251	4.93	66.5	GCA_004683865.2
22	*P. limosus*	JCM 17370	3.91	66.1	GCA_009711185.1
23	*P. litorisediminis*	NBRC 112902	5.37	63.6	GCA_009711205.1
24	*P. luteus*	CFH 10530	3.33	69.6	GCA_004522155.1
25	*P. lutimaris*	CECT 8525	4.21	65.2	GCA_003337565.1
26	*P. nototheniae*	I-41R45	4.60	65.3	GCA_004335005.1
27	*P. onubensis*	1011MAR3C25	4.76	60.3	GCA_003591515.1
28	*P. pantotrophus*	DSM 2944	4.41	67.6	GCA_003633525.1
29	*P. salipaludis*	WN007	3.67	68.6	GCA_002287065.1
30	*P. salsus*	EGI L200073	3.30	65.7	GCA_021556615.1
31	*P. sediminilitoris*	DSL-16	3.64	64.5	GCA_003259195.1
32	*P. sediminis*	CMB17	3.51	66.1	GCA_004310385.1
33	*P. seriniphilus*	DSM 14827	4.20	61.6	GCA_900199195.1
34	*P. solventivorans*	DSM 6637	3.38	68.7	GCA_900142875.1
35	*P. sphaerophysae*	HAMBI 3106	3.36	69.1	GCA_000763805.1
36	*P. subflavus*	GY0581	3.18	65.6	GCA_004310345.1
37	*P. sulfuroxidans*	CGMCC 1.5364	4.04	64.2	GCA_007830335.1
38	*P. suum*	SC2-6	3.25	66.9	GCA_003324675.1
39	*P. tegillarcae*	BM15	3.95	62.1	GCA_002847305.1
40	*P. tibetensis*	CGMCC 1.8925	3.91	68.3	GCA_900102505.1
41	*P. versutus*	DSM 582	5.63	67.6	GCA_003387045.1
42	*P. xiamenensis*	12-3	3.64	63.9	GCA_011308835.1
43	*P. yeei*	ATCC BAA-599	4.43	67.5	GCA_000622145.1
44	*Roseobacter litoralis*	Och 149	4.75	57.2	GCA_000154785.2

Table S2 Performance of EasyCGTree, UBCG, and bcgTree conducting phylogenomic analysis of the genus *Paracoccus*

Note: The trees resulted from the methods 1-4 are corresponding to those in Fig. 2A-D, respectively. *, EasyCGTree and UBCG both used FastTreeMP (a multi-threaded version of FastTree; not available on Windows) for phylogeny inference, which used all the threads available by default; so, the setting of two threads only restricted the other third-party programs in the two pipelines. #, when using 50 threads instead, it will only take 31 min and 1 h 53 min for the methods 2 and 4, respectively.

Methods	1	2	3	4
**Pipeline**	EasyCGTree	EasyCGTree	UBCG	bcgTree
**HMMs**	UBCG	essential	UBCG	essential
**Input**	Proteome	Proteome	Genome	Proteome
**Alignment**	Clustal Omega	Clustal Omega	MAFFT	MUSCLE
**Trimming**	trimAl	trimAl	None	Gblocks
**Alignment length (aa)**				
Raw alignment/genes	31647/90	39704/106	30232/92	38933/107
After trimming	26089 (82.4%)	32805 (82.6%)	/	35469 (91.1%)
**Phylogeny inference**	FastTree	IQ-TREE	FastTree	RaxML
**Threads**	2*	2	2*	2
**Elapsed time**	6.5 min	4 h 44 min#	18 min	12 h 14 min#

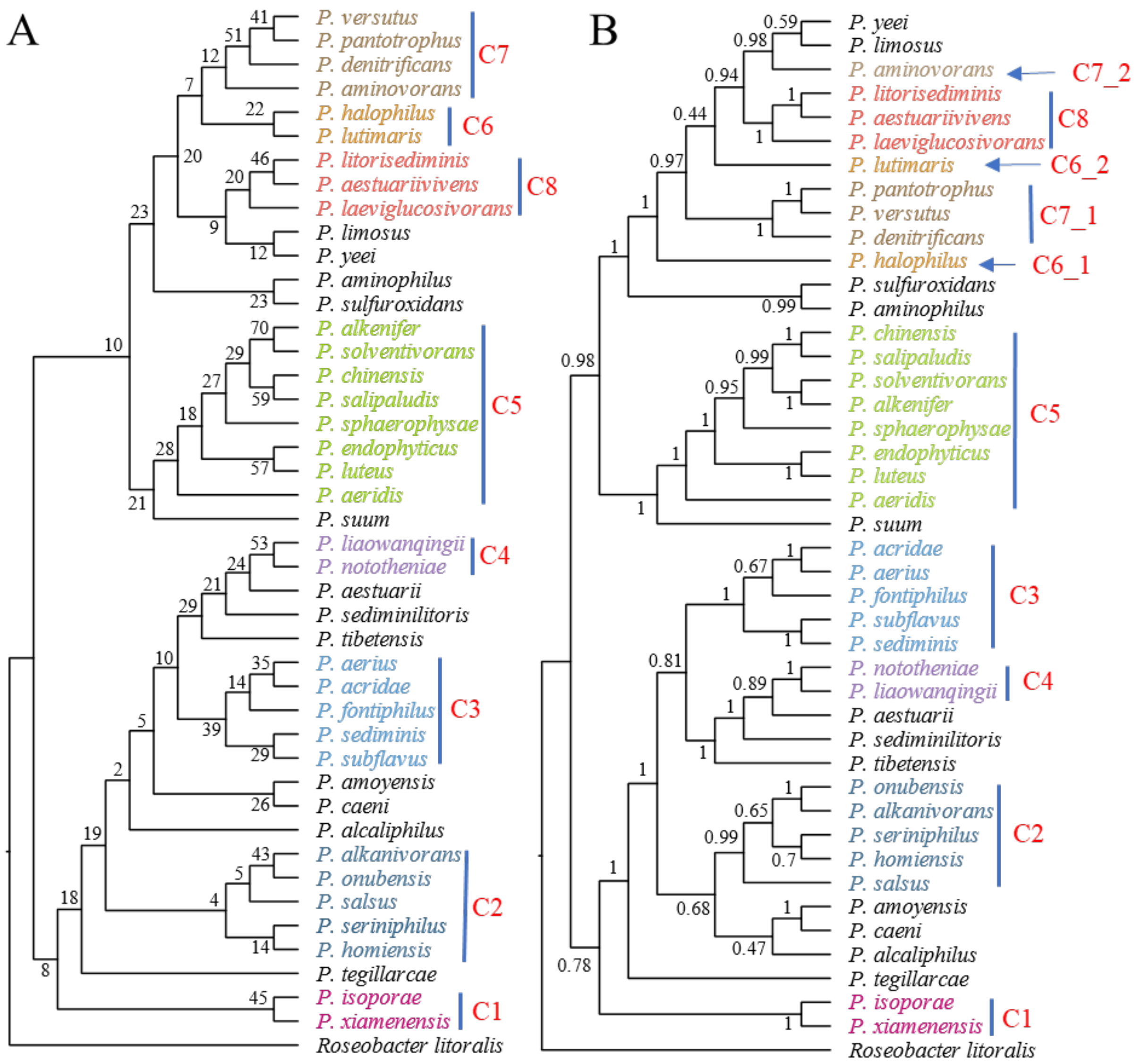

Figure S1 Consensus tree and supertree (ST) of the genus *Paracossus* from the gene set UBCG with EasyCGTree. The Consensus tree was generated based on 79 gene trees of which the genes were universally present in all genomes, while the ST tree based on 90 gene trees (gene families TIGR03625 and TIGR01044 were excluded because of low prevalence). Supporting confidence (A, number of gene tree supporting the node; B, bootstrap) was indicated near the nodes, and Support values 63 (70% out of 90 gene trees) or > 0.7 are considered as significant. Clade (C) corresponding to that in Figure 2 were marked. If species in a clade were not clustered together as in Figure 2, they were indicated using a label like ‘C6_1’.
